# Silicon Oxide Surface Segregation in CO Oxidation on Pd: An in situ PEEM, MS and XPS Study

**DOI:** 10.1007/s10562-012-0955-5

**Published:** 2013-01-08

**Authors:** D. Vogel, Z. Budinska, C. Spiel, R. Schlögl, Y. Suchorski, G. Rupprechter

**Affiliations:** 1 Institute of Materials Chemistry, Vienna University of Technology, Getreidemarkt 9, 1060 Vienna, Austria; 2 Fritz-Haber-Institut der Max-Planck-Gesellschaft, Faradayweg 4-6, 14195 Berlin, Germany

**Keywords:** CO oxidation, Polycrystalline Pd foil, Si segregation, Si oxide formation, Photoemission electron microscopy, X-ray photoelectron spectroscopy

## Abstract

**Abstract:**

The effect of silicon oxide surface segregation on the locally-resolved kinetics of the CO oxidation reaction on individual grains of a polycrystalline Pd foil was studied in situ by PEEM, MS and XPS. The silicon oxide formation induced by Si-impurity segregation at oxidizing conditions, was monitored by XPS and its impact on the global and local (spatially resolved) kinetics of the CO oxidation was determined by MS and PEEM. The results reveal a drastic inhibiting effect of silicon oxide on the Pd reactivity towards CO oxidation, manifested both in the collapse of the global CO_2_ formation rate and in the modified local reactive properties of individual Pd micrograins. The presence of adsorbed oxygen on the Pd surface effectively enhances the silicon segregation to the Pd surface.

**Graphical Abstract:**

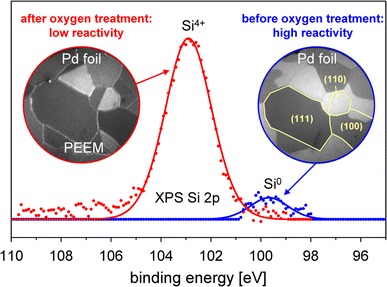

## Introduction

Catalytic CO oxidation on Pt-group metal surfaces is often considered as a simple model reaction of significant practical meaning with respect to pollution emission control. More than three decades ago, pioneering molecular beam experiments of CO oxidation on Pd(111) surfaces performed by Engel and Ertl provided first evidence that the reaction follows the Langmuir–Hinshelwood mechanism [1]. Further applications of the surface science approach to single crystal surfaces revealed the elementary steps of this reaction such as molecular adsorption of CO, dissociative adsorption of oxygen, surface reaction and desorption of carbon dioxide, and culminated in the Nobel Prize awarded to Gerhard Ertl in 2007 [2].

However, at the same time this seemingly simple model reaction was found to be quite complex in case of real catalysts consisting of oxide supported precious metal nanoparticles, and bridging the “materials and pressure gap” between the single crystal surfaces in UHV and real catalysts under ambient conditions is still a challenging task. To overcome the “material gap” between single crystal surfaces and real catalysts, many approaches have been applied, e.g. the investigation of field emitter tips [3], cylindrical single crystals [4] or polycrystalline foils [5–7].

A polycrystalline foil is a particularly suitable model system since it exhibits μm-sized domains of different orientations which are automatically exposed to exactly the same external parameters such as partial pressures and temperature. This means that precise comparative studies of the catalytic behavior of individual (hkl) orientations, such as the role of surface oxide formation can be performed, provided the applied experimental technique is able to read out the information from the μm-sized domains. Our new experimental approach based on photoemission electron microscopy (PEEM) exploits the parallel imaging principle of PEEM and uses the digital analysis of in situ recorded video-PEEM files allowing thus laterally resolved kinetic measurements on a μm-scale [7, 8]. We have already applied this approach to study the local kinetics of the CO oxidation on individual grains of polycrystalline Pt [7, 9] and Pd [10] foil and to directly compare the behavior of reaction–diffusion fronts on differently oriented domains [8, 9].

New experimental developments of surface sensitive techniques applicable under high pressure conditions (i.e. mbar to atmospheric pressure), such as high-pressure scanning tunnelling microscopy (HP-STM), polarization modulation infrared reflection absorption spectroscopy (PM-IRAS) and sum-frequency generation (SFG) have provided insights to catalytic reactions such as CO oxidation under more realistic catalytic conditions (bridging the “pressure gap” [11–16]). In some studies, (surface) oxide formation was observed in parallel with an activity increase and a Mars-van-Krevelen like mechanism has been proposed for the CO oxidation reaction on Pt and Pd surfaces at elevated pressures [16, 17]. Despite an intensive debate about the reaction mechanism and about the nature of the most active phase in CO oxidation [18, 19], the Langmuir–Hinshelwood mechanism seems still to predict well the kinetics of the CO oxidation reaction on Pt-group metals at both low and high pressures [12]: the O-covered metal surface responsible for the high catalytic activity in UHV, appears also as the most active phase at elevated pressures, unless oxide formation initiates the deactivation, especially for Pd and Rh surfaces.

Despite of the intensive studies of the role of palladium oxides in CO oxidation on Pd [20], much less attention is directed to the role of “non-palladium” oxides on the Pd surface in the CO oxidation. Recently it was shown, that such “stranger” oxides present on the platinum-metal surface can significantly influence the reaction, even at small oxide coverages [21, 22]. It is also known that impurities commonly present in commercially used catalysts, such as Si, might form surface oxides under oxidizing atmosphere [23], but, to our knowledge, aimed studies of such oxides under reaction conditions are very scarce. In the present contribution we apply PEEM combined with in situ x-ray photoelectron spectroscopy (XPS) and mass-spectrometry (MS) to study the role of the surface oxides formed by Si impurities in the kinetics of the CO oxidation on Pd.

## Experimental

**Fig. 1:**
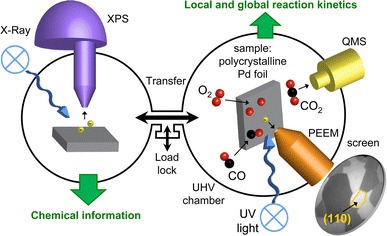
Configuration of the experimental setup and scheme of the experiment. The simultaneous monitoring of the ongoing CO oxidation by PEEM and MS provides information on kinetic transitions on the whole sample (global MS measurements) and on the individual grains of a polycrystalline Pd foil (local PEEM measurements). The XPS analysis provides data on the chemical composition of the samples in UHV and under the same gas atmosphere as in PEEM

The experiments were performed in a UHV system consisting of two independently operated chambers connected with each other by a sample transfer line, thus allowing a common reactive gas atmosphere in the 10^−4^–10^−9^ mbar range. The “microscopy” chamber is equipped with a PEEM (Staib Instruments), an MS (MKS Instruments), a LEED system (Omicron), a high purity gas supply system (O_2_: 99.999 %, CO: 99.97 %) and sample preparation facilities for cleaning the sample by argon ion sputtering and subsequent annealing. The “spectroscopy” chamber is equipped with an XPS-system (Phoibos 100 hemispherical energy analyzer and XR 50 twin anode X-ray source, SPECS). The general configuration of the experimental setup and a scheme of the experiment are shown in Fig. 1.

The investigated sample, a 10 × 10 mm^2^ polycrystalline Pd foil (AlfaAesar, 99.9 % purity), consists mainly of [100]-, [110]- and [111]-oriented grains which were formed after annealing the foil for several hours at 1100 K in UHV. The sample temperature was measured by a NiCr/Ni thermocouple spot-welded to the front side of the Pd sample. Prior to catalytic measurements, the surface was repeatedly Ar^+^ sputtered and annealed at 1073 K.

The principle of the local kinetic measurements by PEEM has been described in detail before [7–9] and is therefore summarized here only briefly (see also Fig. 1). The CO oxidation reaction on the Pd foil is monitored simultaneously by MS and by PEEM, whereby MS provides the global CO_2_ rate produced by the whole polycrystalline sample. PEEM, in turn, provides the laterally resolved kinetic information from the individual grains.

The PEEM image which is formed by photoelectrons created by UV light illumination of the sample (with a deuterium discharge lamp, E_max_ ~6.5 eV) reflects the lateral distribution of the local work function across the sample. This allows for differentiation between grains of different surface orientation and between different adsorbates by correlation of the local image intensity with the work function values of the corresponding clean and adsorbate-covered single crystals [7]. The PEEM-image is recorded in situ with a high-speed CCD-camera (Hamamatsu), so the intensity of a selected region-of-interest, i.e. one particular grain, can be monitored during a typical CO pressure scan. The XPS analysis provides data on the chemical composition of the samples in UHV or under the same gas atmosphere as in PEEM.

## Results and Discussion

### Global and Local Reaction Kinetics

**Fig. 2:**
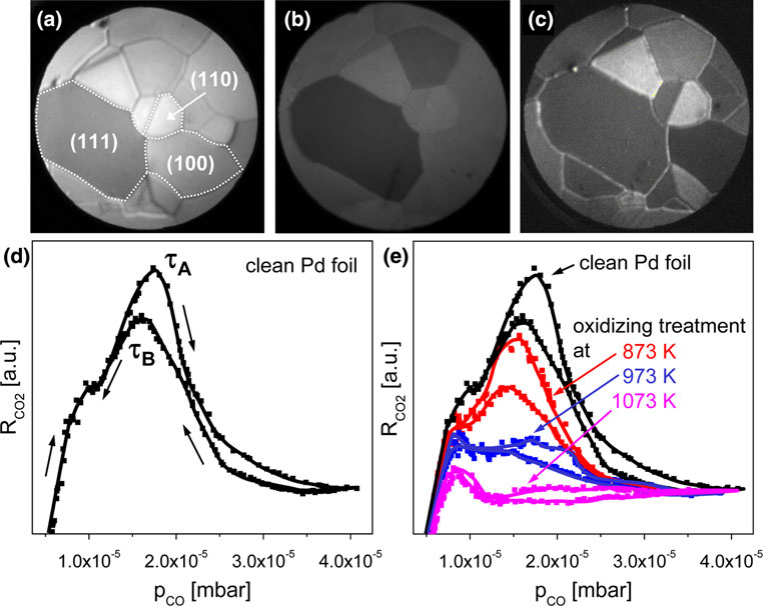
CO oxidation on polycrystalline Pd foil. (**a**) PEEM image of the clean (Ar^+^ sputtered and annealed) Pd surface, three low index domains are exemplary indicated, (**b**) the same surface, but oxygen covered, (**c**) the same surface after oxidizing treatment at po_2_ = 5 × 10^−6^ mbar and T = 973 K, (**d**) global CO_2_ reaction rate for a clean Pd surface recorded by MS during an isothermal (T = 473 K) cyclic CO pressure scan at constant oxygen pressure of 1.3 × 10^−5^ mbar. Kinetic transitions τ_A_ and τ_B_ from the catalytically active to the inactive steady state and vice versa are indicated, (**e**) the same as in (**d**) but for the oxygen treated surface (po_2_ = 5 × 10^−6^ mbar, T = 873, 973, 1073 K, treatment duration 15 min for all temperatures). A CO_2_ reaction rate curve for the clean surface is also shown for comparison

To correlate the kinetic transitions in CO oxidation reaction on individual grains on the polycrystalline Pd foil with the averaged (global) kinetics of the whole sample, the orientation of the individual grains was established first, and global kinetic studies in the bistability range of CO oxidation were performed. The results are summarized in Fig. 2: the upper row (Fig. 2a–c) shows PEEM-frames of the clean (Ar^+^ sputtered and annealed) Pd foil (Fig. 2a), the same surface covered by adsorbed oxygen (Fig. 2b) and again the same area but after oxygen treatment at 973 K and 5 × 10^−6^ mbar (Fig. 2c). In Fig. 2a three individual [110]-, [100]- and [111]-oriented grains are indicated, whose crystallographic orientation has been determined by the differences in contrast, as described in detail in Ref. [7]. The lower plots in Fig. 2 show the global (MS-measured for the whole sample) CO_2_ reaction rate recorded during an isothermal cyclic CO pressure scan at constant oxygen pressure of 1.3 × 10^−5^ mbar for the clean, i.e. sputtered and annealed Pd surface (Fig. 2d), and for the same surface which was additionally treated by oxygen at po_2_ = 5 × 10^−6^ mbar for 15 min at three different temperatures of 873, 973 and 1073 K, also in comparison with the clean Pd surface (Fig. 2e).

Typically for CO oxidation on Pd, the surface in the high reactivity steady state (predominantly oxygen-covered), exhibits an almost linear increase of the CO_2_ production rate with increasing CO pressure, till a kinetic transition from the high reactivity state to a low reactivity state occurs (Fig. 2d). This is the result of the collapsing oxygen adsorption due to the CO-induced poisoning (inhibition of dissociative oxygen adsorption, [2]) of the Pd surface. The corresponding transition point is termed τ_A_ whereas the point of the reverse transition is called τ_B_ (Fig. 2d). Because of the effective blocking of adsorption sites for oxygen by CO the reverse transition τ_B_ from the inactive to the active state occurs at a lower CO partial pressure than the transition τ_A_, resulting in the hysteresis in the CO_2_ production rate as seen in Fig. 2d for the sputtered and annealed Pd surface and in Fig. 2e for the Pd surface treated at 873 K in oxygen at 5 × 10^−6^ mbar. Such hysteresis-like behavior of the CO oxidation reaction is called bistability since two states of the reaction system are possible at the same parameter set depending on the system’s prehistory (for details see refs. [21, 22]).

For the Pd surface treated by oxygen at 873 K the CO_2_ rate appears considerably lower, but the hysteresis is still present (Fig. 2e). Oxygen treatment at higher temperatures causes further decrease of the CO_2_ rate (e.g. for 973 K, Fig. 2e) and even a disappearance of the hysteresis (see the 1073 K curve in Fig. 2e).

**Fig. 3:**
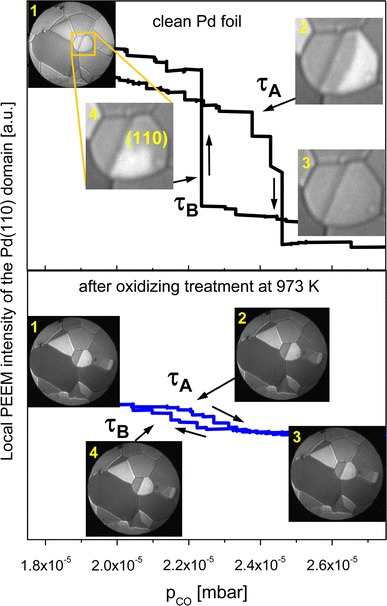
Laterally-resolved kinetics of CO oxidation on individual grains of the Pd foil, (**a**) local intensity of the PEEM image for the Pd(110) domain recorded during the same cyclic CO pressure scan as in Fig. 2d. The kinetic transitions τ_A_ and τ_B_ are much more pronounced than in the global MS curve (Fig. 2d). The PEEM frames correspond to the reaction stages indicated in the PEEM intensity curve, (**b**) the same as in (**a**) but after the oxidizing oxygen treatment at 973 K. The τ_A_ and τ_B_ points are almost indiscernible

For the laterally resolved kinetics of the CO oxidation, the video-PEEM sequences (recorded simultaneously with the MS monitoring) were analyzed. Figure 3a shows an example of such analysis where the *local* PEEM intensity for a particular domain, in the present case a Pd(110) domain, is shown in analogy to the *globally* MS-measured CO_2_ rate in Fig. 2d. The local data result in a clear hysteresis where two sharp drops/jumps of the PEEM intensity are visible. Analogue to the changes in the overall CO_2_ reaction rate in dependence of the CO partial pressure as measured by MS, the drops/jumps in the PEEM intensity represent kinetic transitions from one reactivity state of the system to another, but measured locally for one particular grain of interest. The reason for the much more pronounced local transitions observed by PEEM (in comparison to MS) lies in the averaging effect of the MS measurements: the kinetic transitions of the differently oriented grains occur at different CO pressures, the corresponding drops/jumps in the R_CO2_ curve overlap, thus smearing out the global τ_A_ and τ_B_.

Again, similarly as in the global case, the treatment of the polycrystalline Pd surface with oxygen at elevated temperature (e.g. at 973 K as is shown in Fig. 3b) results in the collapse of the hysteresis loop, the local τ_A_ and τ_B_ transitions are smeared out and it is not possible to associate the transitions with particular CO pressure values as in the case of the clean Pd surface (Fig. 3a). The reason for this behavior will be discussed below, together with the XPS data.

### XPS Results

**Fig. 4:**
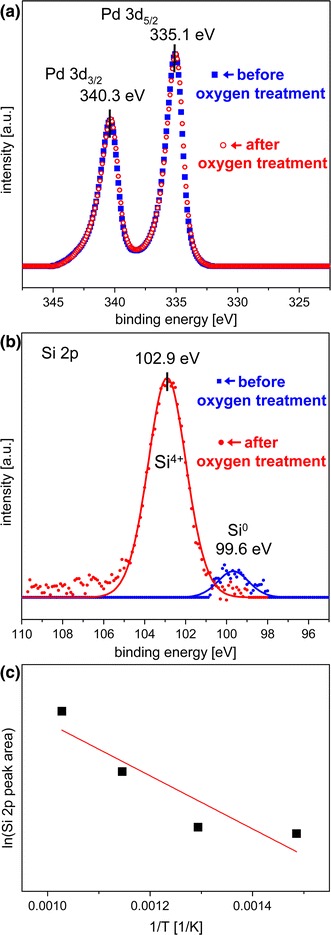
Silicon oxide formation on Pd surface. (**a**) Pd 3d XPS spectra before and after oxidizing oxygen treatment at 973 K, (**b**) the same but for the Si 2p spectra, (**c**) Arrhenius plot

To explain the observed reaction behavior, an XPS-analysis of the Pd foil has been performed before and after the oxidizing oxygen treatment at constant oxygen pressure of 5 × 10^−6^ mbar and temperatures between 673 and 973 K. The XPS results are summarized in Figs. 4a–c, in which the high-resolution spectra for the Pd 3d region (Fig. 4a), for the Si 2p region (Fig. 4b) and the evolution of the Si 2p signal with surface oxidation temperature (Fig. 4c) are shown. The Pd 3d spectra demonstrate that no significant formation of palladium oxide has been observed under the present conditions. In turn, the Si 2p spectra reveal a remarkable SiO_2_ formation on the Pd surface upon the oxidizing treatment at elevated temperatures. Apparently, the presence of oxygen stimulates the diffusion of bulk dissolved Si from the near-surface regions towards the surface and thus enhances the segregation of Si to the Pd surface (compare the Si^0^ and the Si^4+^ 2p signals, measured for the Si segregated without and with oxygen atmosphere). As for any diffusion related process, the segregation limited SiO_2_ formation rate appears to be strongly temperature dependent, as demonstrated in Fig. 4c. An attempt to apply an Arrhenius plot provides an effective activation energy of 0.27 eV, a value which is significantly lower than known values of about 2 eV for thermal Si oxidation [24, 25]. It is clear, however, that in the present case of a submonolayer SiO_2_, the Deal-Grove model [24] based on the assumption that Si oxidation proceeds by the transport of molecular oxygen from the ambient to the Si/SiO_2_ interface through already oxidized Si layers does not apply. In our case, the rate limitation does not result from the oxygen diffusion, but from the silicon transport from the Pd subsurface region to the surface. Thus, the activation energy values typical for the oxygen diffusion limited process are not expected. Recently, unusually small activation energy values (of e.g. 0.13 eV [25] or even lower [26]) were observed for the oxidation procedure where the oxygen diffusion limit was lifted by using atomic oxygen for Si oxidation.

Generally, experimental observations are known, which show that the presence of oxygen (even in the 10^−6^ mbar range) increases the mobility of Si in Pt-metals causing the precipitation of SiO_x_ at the surface [27]. The enhanced Si segregation in the presence of oxygen can be rationalized considering energetic arguments: the higher bonding strength of Si on an oxygen covered Pd surface provides an energetic sink for Si atoms due to the chemical potential gradient, similarly as it occurs e.g. in an enrichment of alkali atoms on the oxygen covered surface in CO oxidation on Rh [28] or hydrogen oxidation on Rh [29, 30], or in the formation of concentration patterns of electropositive adsorbates as a result of up-hill diffusion in two-dimensional first order phase transitions under reaction-free conditions [31, 32]. Recently, DFT calculations were performed to identify the stable Si–O bonding structures on the Pd(111) and Pt(111) surfaces [33], where a variety of stable intermediates with stoichiometries between SiO_2_ and SiO_3_, depending on the coverage, were found. This might explain the inhibiting effect of the SiO_x_ growth on the PdO_x_ formation observed in the present experiments, again due to the energetic arguments which favor the Si rather than the Pd oxidation.

The easiest interpretation of the inhibiting role of SiO_2_ in the CO oxidation reaction would be that SiO_2_ simply blocks a part of the active reaction sites on the Pd surface but does itself not participate in the reaction. However, the drastic decrease in the CO_2_ formation rate for a rather small coverage of SiO_2_ (just a fraction of a monolayer, as follows from the Pd 3d and Si 2p signal relation) requires an additional explanation. It is known that oxides on the Pt-group metals form monolayer islands at the initial growth stage [22, 34]. The boundary lines between the oxide islands and the remaining “oxide-free” metal surfaces exhibit, due to the local electron density jump from the metal to the oxide surface, different electronic properties which may nanometer-wide extend the promoting effect along the free metal surface around the islands [22].

The promoting effect on the reaction observed in the case of CeO_x_ islands, which is due to the additional oxygen supply [22], cannot be expected for SiO_2_ because of the poor redox properties of SiO_2_ and thus the absence of an oxygen storage capacity. Whereas the refilling of the oxygen vacancies in reducible oxides such as CeO_x_ stimulates the oxygen dissociation on the oxide-island adjacent surface sites, this is not the case for SiO_2_. In turn, the spillover of CO from the SiO_2_ islands (a weakly bound mobile CO layer can be formed on the SiO_2_ surface [35]) supports the interpretation that the regions around the SiO_2_ islands must be rather inactive for the CO oxidation.

## Summary

A combined PEEM-MS-XPS approach allows us to study the in situ kinetics of surface reactions under control of the surface chemical composition of the sample, also under reactive atmospheric conditions in the 10^−5^ mbar range. This also allows to trace reaction induced compositional changes of the sample surface, e.g. as the result of segregation effects.

In the present study silicon oxide formation, resulting from Si-impurity segregation on the polycrystalline Pd-foil at oxidizing conditions in the temperature range between 873 K and 1073 K, was monitored by XPS and its impact on the global and local (spatially-resolved) kinetics of the CO oxidation was determined by MS and PEEM. The results reveal a drastic inhibiting effect of silicon oxide on the Pd reactivity towards CO oxidation, manifested both in the collapse of the global CO_2_ formation rate (as measured by MS) and in the modified local reactive properties (as observed by PEEM) of individual Pd micrograins. The presence of adsorbed oxygen on the Pd surface effectively enhances the silicon segregation to the Pd surface, due to energetic reasons.
